# Retraction: Intersecting paths: Corporate and green innovation in Chinese firms—A panel cointegration analysis

**DOI:** 10.1371/journal.pone.0331587

**Published:** 2025-09-04

**Authors:** The PLOS One Editors

The *PLOS One* Editors retract this article [1, corrected in 2] because it was identified as one of a series of submissions for which we have concerns about potential manipulation of the publication process, peer review integrity, and authorship. These concerns call into question the validity and provenance of the reported results. We regret that the issues were not identified prior to the article’s publication.

RMAZ did not agree with the retraction. ZJL and DH either did not respond directly or could not be reached.

## References

[1] Liu Z, Hou D, Zahid RMA. Intersecting paths: Corporate and green innovation in Chinese firms-A penal cointegration analysis. PLoS One. 2024;19(1):e0295633. doi: 10.1371/journal.pone.0295633 38232076 PMC10793966

[2] Liu Z, Hou D, Zahid RMA. Correction: Intersecting paths: Corporate and green innovation in Chinese firms-A penal cointegration analysis. PLoS One. 2025;20(1):e0318610. doi: 10.1371/journal.pone.0318610 39874359 PMC11774364

